# Application of the shear wave elastography in the assessment of carotid body tumors: A preliminary study

**DOI:** 10.3389/fonc.2022.1053236

**Published:** 2023-01-06

**Authors:** Xiaoyan Zhang, Yuehong Zheng, Jianchu Li, Bo Zhang

**Affiliations:** ^1^ Department of Ultrasound, State Key Laboratory of Complex Severe and Rare Diseases, Peking Union Medical College Hospital, Chinese Academy of Medical Sciences and Peking Union Medical College, Beijing, China; ^2^ Department of Vascular Surgery, State Key Laboratory of Complex Severe and Rare Diseases, Peking Union Medical College Hospital, Chinese Academy of Medical Sciences and Peking Union Medical College, Beijing, China; ^3^ Department of Ultrasound, China-Japan Friendship Hospital, Beijing, China

**Keywords:** carotid body tumor (CBT), shear wave elastography (SWE), ultrasonography, elasticity, shear wave velocity

## Abstract

**Objectives:**

To evaluate the elasticity of carotid body tumors (CBTs) by two-dimensional shear wave elastography (SWE).

**Methods:**

22 pathologically or clinically confirmed CBTs in 16 patients were scanned by SWE. The maximum elasticity value (Emax) and its standard deviation (SDmax) in kPa and m/s for CBTs were obtained by placing a round ROI (2-3 mm) on the stiffest region of the CBTs. Elasticity value was compared between hard and soft groups at manual palpation, benign and malignant groups and among three Shamblin types. The area under the receiver operating characteristic curve (AUC) analysis was performed to evaluate the performance of SWE in the malignancy prediction of CBTs. Sensitivity, specificity and accuracy were calculated. The cut-off value was obtained by using the Youden index.

**Results:**

There were 19 benign CBTs and 3 malignant CBTs. Emax (kPa and m/s) and SDmax (kPa) were significantly higher in the hard group than in the soft group at manual palpation (*P*<0.05); The distribution of Emax in kPa and m/s and SDmax in kPa were different in the three Shamblin types (*P*<0.05), Emax (kPa and m/s)increased from shambling I to Shambling II and Shambling III; Emax (kPa and m/s) were significantly higher in the malignant CBTs than in the benign ones (*P*<0.05). Emax in kPa and m/s had the similar AUC value (AUC=0.947, *P*=1.0000) for the prediction of malignant CBTs. Emax in kPa with the cut-off 124.9kPa showed a sensitivity of 100.0%, specificity of 94.7%, and an accuracy of 95.5% (*Z*=8.500, *P*<0.0001); Emax in m/s with the cut-off 5.9m/s showed a sensitivity of 100.0%, specificity of 89.5% and an accuracy of 90.9% for the prediction of malignant CBTs (*Z*=9.143, *P*<0.0001).

**Conclusions:**

Quantitative analysis of SWE obtained the good performance in the elasticity assessment of CBTs.

## Introduction

Carotid body tumors (CBTs) are slowly growing paragangliomas (PGL) arised from cells of neural crest origin that most often located at the adventitia of common carotid artery bifurcation. CBTs represent about 45-75% of head and neck PGL, and 4%-15% of CBTs have a malignant tendency to display invasive behavior (vascular invasion, capsular invasion, and/or extension into the surrounding tissue) or develop distant metastasis (1–3).

The majority of CBTs are asymptomatic and initially noticed by inspection and manual palpation of neck swelling during the physical examination or as incidental findings on radiological imaging studies. Elasticity is the tendency of tissue to resist deformation with an applied force, or to resume its original shape after removal of the force (4). Manual palpation is a clinical diagnosis method utilized by physicians to acquire elasticity information of an organ using the sense of touch (5). It can be used to check whether there is a mass in lateral neck and evaluate its elasticity. Nevertheless, it has several limitations, such as lack of sensitivity for the deep-situated or small lesions, also, it is subjective and highly dependent on the physician’s experience and skill. It was reported that 72% of the CBTs were discovered on clinical or self-examination (6). Conventional Ultrasound (US), as a non-invasive, non-radiation imaging modality, can visualize the target lesion through real-time scanning and give detailed information about the lesion. However, the elasticity information of lesions is not available with conventional US. Applications of shear wave elastography (SWE) will be complementary and help physician to better define neck masses. Shear waves are generated by applying mechanical vibration or pressure or by applying acoustic pressure, which enables objective measurement of shear wave propagation velocity measurements. Benign lesions tend to be soft, while malignant lesions tend to be relatively stiffer. The propagation velocity differs significantly depending on the tissue stiffness. The measured shear wave velocity (SWV) (m/s) is converted to the Young’s modulus (elastic modulus, kPa) using the formula: E= 3 · ρ · V^2^. In recent years, SWE has been widely used in diffuse liver diseases, stiffness evaluation of carotid atherosclerotic plaques and in the identification of benign and malignant tumors, effectively improving diagnostic confidence (7–13).

Malignant PGL tended to show mitotic figures, pleomorphism, necrosis, vascular invasion and large amount of collagen fiber bands dividing the tumor cell (14, 15). Presence of extensive invasion of adjacent organs and distant metastasis is one of the manifestation of malignancy (16). Predicting malignant disease in patients presenting with primary CBTs without metastases is difficult. While surgery resection is commonly curative in benign PGL, the prognosis for malignant PGL remains poor, the 5-year relative survival rate was 59.5% (76.8% for regionally confined carcinoma and 11.8% for distant metastasis) (17). Early diagnosis and distinguishing malignant from benign CBTs are necessary for appropriate treatment decision and adequate follow-up. Conventional US, computed tomography (CT) and magnetic resonance imaging (MRI) are sensitive to localize the primary tumor. However, these imaging modalities are limited in discriminating between benign and malignant CBTs. SWE approach in CBTs has not been investigated in clinical practice. Therefore, the purpose of this study was to explore the SWE approach in the elasticity assessment of CBTs.

## Materials and methods

### Patients

Ethical approval was obtained from the institutional review board and informed consent was obtained from all patients for participating in this prospective study. The following inclusion criteria were applied: 1) the presence of lateral neck masses; 2) CTA performed and proved to be CBTs according to typical radiographic characteristics (18); 3) Patients for whom conventional US and SWE screening were performed. From December 2017 to April 2018, 21 consecutive patients with lateral neck masses who were referred to our hospital underwent Conventional US and SWE examination. Of these patients, five patients with 2 schwannomas, 1 carotid bifurcation hemangioma, 1 branchial cleft cyst and 1 squamous cell carcinoma of cervical muscle spaces confirmed by biopsy pathology or postoperative histopathology were excluded. 16 patients with 22 CBTs who were referred to Vascular Center for further evaluation were enrolled in the current study.

### Conventional US and SWE examination

Conventional US and SWE examinations were performed with the Aplio500 US machine equipped with an 14L5 transducer (Canon [formerly Toshiba] Medical Systems Corporation, Otawara, Japan) by one radiologist (XZ with >10 years of experience in US and >2 years of experience in elastography). All patients were scanned in a supine position to obtain the transverse and longitudinal US images for the target lesions on the neck. First, US images were obtained, including B-mode and color Doppler images. The tumor size, shape, echogenicity, margin and vascularity were evaluated by US. Subsequently, SWE examinations were conducted with the same transducer at the transverse images of the CBTs. Two-dimensional shear wave elastography integrated with the Amplio500 US machine was used for the calculation of the SWE. The transducer was applied with an adequate amount of contact gel and kept vertical to the target lesion with pressure as slight as possible. SWE images were obtained with the patients holding breath for a few seconds under “One Shot Scan” mode. The interval between two performances is 5 seconds. SWE images can be viewed and switched among three options after image frozen: speed mode (shear velocity, m/s, range 0.5-8.0m/s), elasticity mode (kPa, range 0-180kPa) and propagation mode (arrival time contour). The SWE images in three modes were displayed in semi-transparent 2D-color imaging, which were overlaid on the B-mode image. The elastic modulus or SWV is displayed by the gradual colors with increasing stiffness revealed in ascending order of blue, green, yellow and red. Regions which are not color-coded on the elasticity images reflect that no shear wave is detected. In the propagation mode, it displays arrival time contour with the shape of contour lines. In areas where the contour lines are parallel, the shear waves propagate properly and the reliability of the obtained data is high. In areas where the contour lines are distorted and not parallel to one another, the reliability of the obtained data is low. In other words, the reliability of the data can be verified by observing the contour lines. The intervals between the displayed contour lines are wider in stiff tissues and narrower in soft tissues. The SWV and elasticity values can be measured quantitatively by placing an ROI in both speed mode and elasticity mode. The measured SWV (m/s) can be converted to the Young’s modulus (kPa) using the formula: E = 3 · ρ · V^2^ where E is the Young’s modulus (kPa), ρ is density of tissue (kg/m^3^) and V is the SWV (m/s). In this study, the ROIs were placed on the stiffest part of the CBTs. The system automatically calculated the maximum elasticity (Emax) and its standard deviation (SDmax) in kPa and m/s. SWE is carried out for two times and the average value is used.

### Shamblin type

CBTs were classified into 3 types by Shamblin and colleagues. A Shamblin I CBT is small and localized, a Shamblin II surrounds vessels or partially encloses them, and a Shamblin III is large and fully encloses adjacent vessels (19).

### Computed tomography angiography

All patients underwent CTA tests prior to admission or during hospitalization. CTA scan showed a soft tissue mass with contrast agent enhancement at unilateral or bilateral carotid bifurcation, spraying the angle of carotid bifurcation.

### Identification of malignant CBTs

The malignancy was identified based on regional lymph node metastasis and distant metastasis of nonneuroendocrine tissue (most commonly include cervical lymph nodes or distant sites such as the lung, bone, liver and skin) combined with high heterotypical histopathologic presentation (17, 20, 21).

### Manual palpation

Manual palpation of the CBTs was carried out and recorded by the attending physician of vascular center during the physical examination after admission, and the stiffness at manual palpation was divided into two groups: soft group and hard group. The Emax and SDmax expressed in kPa and m/s of CBTs in the soft and hard group was examined and compared.

### Surgery

Of the 16 CBT patients enrolled in the study, 11 patients with 15 CBTs underwent surgical therapy and no surgical mortality or severe morbidity was observed (bilateral CBTs resection in 4 patients, unilateral tumor resection in 2 patients with bilateral tumors with the contralateral tumors regular scanning); 3 patients chose regular monitoring because of small lesions suitable for waiting and scanning strategy or patients’ refusal to be operated due to concerns about surgery; 2 patients with 2 malignant CBTs who were found to have invasion of surrounding tissues and systemic distant metastasis did not undergo surgery.

All patients have referred to the vascular center for periodic monitoring, regardless of whether the tumor was removed surgically or not. All patients were followed up through medical record after 5 years from the first outpatient consultation. No regional lymph node metastasis and distant metastasis of nonneuroendocrine tissue were observed in the benign groups.

### Statistical analysis

The quantitative data are presented as the means and standard deviations. The Shapiro-Wilk test was used to determine the presence of a normal distribution. The unpaired t test was used to evaluate differences between the two groups for parametric data. Categorical variables are presented as number and frequency, and analyzed using the χ^2^ test. The intra-operator consistency of quantitative SWE were evaluated with the intra-class correlation coefficient. The Mann-Whitney U-test (two variables) and the Kruskal-Wallis test (three or more nominal variables) were used to evaluate differences between two or more groups for non-parametric data. Receiver operating characteristic (ROC) curve analysis was performed and the area under the ROC curve (AUC) analysis was performed to evaluate the performance of SWE in the malignancy prediction of CBTs. Sensitivity, specificity, positive predictive value (PPV), negative predictive value (NPV) and accuracy were calculated. The cut-off value of each parameter was obtained by using the maximum Youden index. Statistical analyses were performed with SPSS software version 20.0 (IBM, Armonk, NY, USA). Differences with *P*<0.05 were considered statistically significant.

## Results

### Clinical and US characteristics of CBTs

A total of 16 patients (mean age: 41years, range 24-63years) with 22 CBTs were screened by conventional US and SWE. Demographics and clinical findings were shown in **Table 1**. The most common clinical presentation was an asymptomatic mass of the lateral cervical region (100%); two patients accompanied with hoarseness for 4 years and 1 year respectively. The mean altitude of all the patients lived was 722.8m (range 6.6-2261.2m). 43.7% (7/16) patients lived in areas above 722.8m above sea level. The mean living altitude of the patients with bilateral CBTs was 1127.3m (range 52-2261.2m). Two patients were brothers who came from plateau area with an altitude of 2261.2m, both with bilateral CBTs.

**Table 1:** Clinical characteristics of CBT lesions.

Clinical information	Numbers (%)
Gender	
Male	11 (68.8)
Female	5 (31.2)
Tumor Location	
Left	4 (25.0)
Right	6 (37.5)
Bilateral	6 (37.5)
Tumor number	22
**Family history**	2 patients (12.5)
**Shear wave elastography**	22
**Surgical treatment (n)**	15 lesions in 11 patients
Diagnosis	
Benign	19 (86.4)
Malignant	3 (13.6)

**Figure 1:**
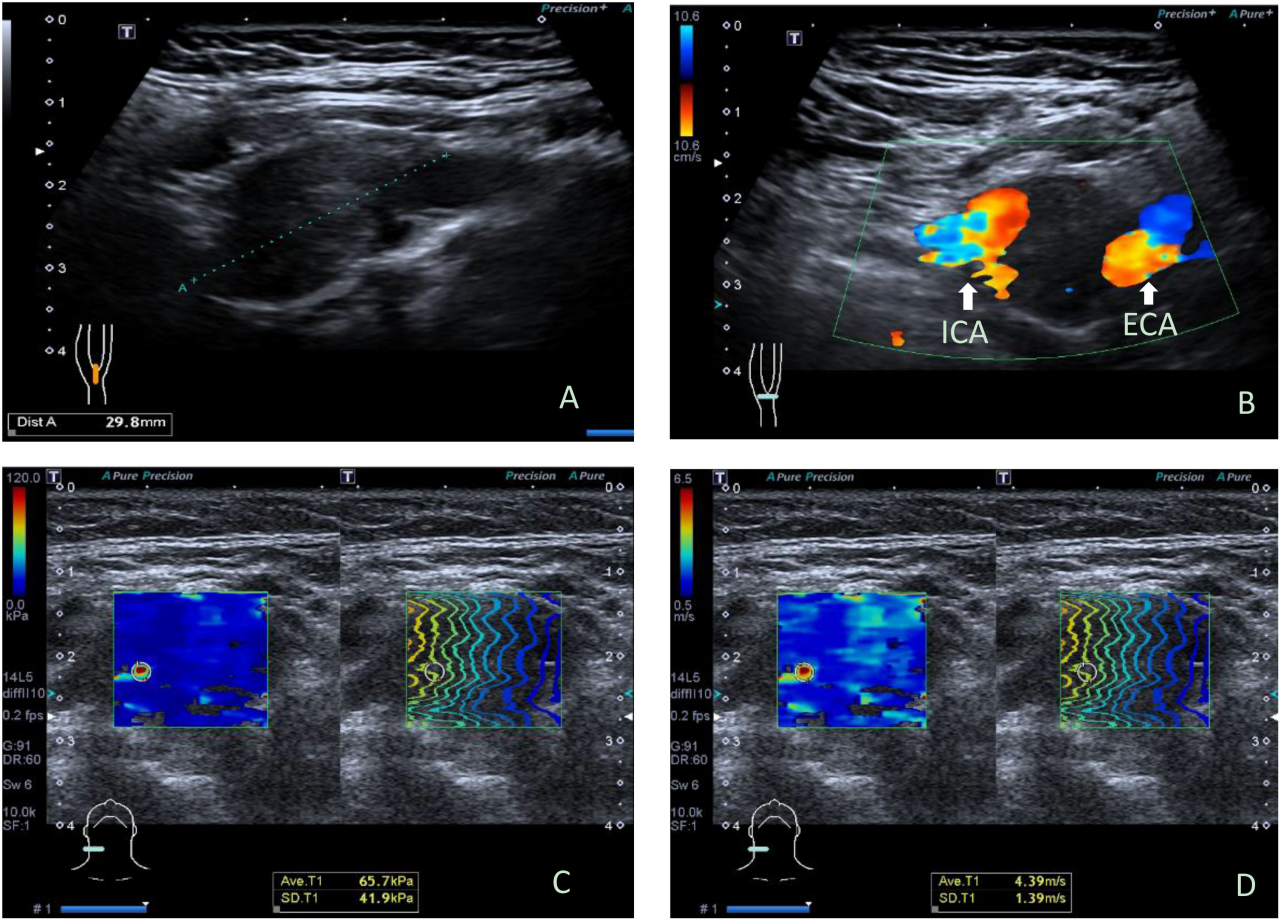
Ultrasonographic images in a 34y female with benign carotid body tumor. **(A)** Conventional longitudinal B-mode sonogram showed an oval hypoechoic mass with clear boundary located at the right carotid bifurcation, **(B)** Color Doppler flow imaging on transverse image showed the carotid body tumor partially encloses internal carotid artery and external carotid artery, splaying the carotid bifurcation. **(C)** The parameters of SWE on elasticity mode were as follows: Emax: 65.7kPa, SDmax: 41.9 kPa; **(D)** The parameters of SWE on speed mode were as follows: Emax: 4.39m/s, SDmax: 1.39cm/s. ICA, internal carotid artery; ECA, external carotid artery; SWE, shear wave elastography.

**Figure 2:**
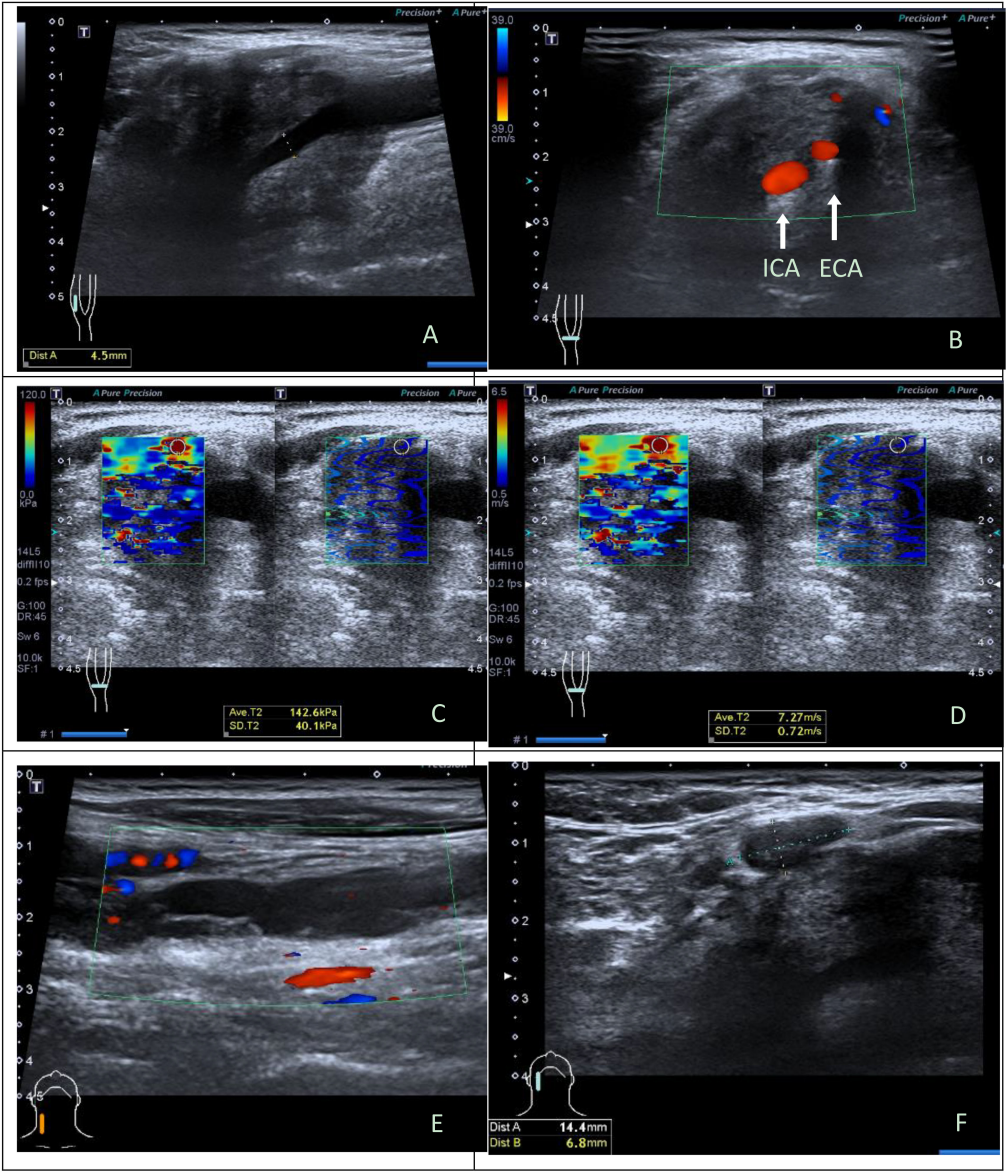
Ultrasonographic images in a 43y male with malignant carotid body tumor. **(A)** Conventional longitudinal B-mode sonogram showed an irregular hypoechoic mass with blurred boundary located at the right carotid bifurcation. **(B)** Color Doppler flow imaging on transverse image showed the carotid body tumor was large and fully enclosed internal carotid artery and external carotid artery. **(C)** The parameters of SWE on elasticity mode were as follows: Emax, 142.6kPa, SDmax, 40.1kPa; **(D)** The parameters of SWE on speed mode were as follows: Emax, 7.27m/s, SDmax, 0.72cm/s. **(E)** Hypoechoic band structure can be seen at the eccentric part and below of the tumor body on longitudinal sonogram, a few blood flow signals can be seen at the edge and inside it on color Doppler flow imaging; **(F)** A lymph node could be seen adjacent to the tumor, with the interruption of lymph node capsule continuity on transverse sonogram. Intraoperatively, it was confirmed that hypoechoic band structure was the right internal jugular vein invaded and wrapped by the tumor. Histopathological results: CBT with degeneration, involving peripheral adipose tissue, lymph node capsule and internal jugular vein. Immunohistochemistry: Melan-A (-), AE1/AE3 (-), CgA (+), Ki-67(index 10%), S-100(+), α-inhibin (-). ICA, internal carotid artery; ECA, external carotid artery; SWE, shear wave elastography.

There were 19 benign CBTs (**Figure 1**) and 3 malignant CBTs. Three patients (18.8%) underwent imaging modalities including conventional US, SWE, CTA and somatostatin receptor positron emission tomography/computed tomography (PET/CT), two patients of them were diagnosed with malignant CBT with regional and multiple distant metastases (Pharynx, larynx, cervical lymph node, Lung, bone, rectum, sigmoid colon, etc.) and did not undergo surgical treatment. Another patient suspected of malignancy by imaging modalities had no distant metastasis and underwent surgery (**Figure 2**).

Tumor size ranged from 1.3 to 10 cm (mean size 3.7 ± 1.6 cm). The mean size of Shamblin I, II, and III tumors were 1.8, 3.6 and 6.7cm respectively. The size of CBTs was positively correlated with shambling type (*r*=0.658, *P*<0.001), Emax in kPa (*r*=0.525, *P*=0.012) and Emax in m/s (*r*=0.485, *P*=0.022). Patients in the malignant group experienced a longer course than the patients in the benign group (median course: 10 years VS 0.5year, *t*=-2.035, *P*=0.042).

### Manual palpation

All the three malignant CBTs were hard texture at manual palpation, and among 19 cases of benign CBTs, 12 were soft and 7 were hard texture at manual palpation, there was significant difference at manual palpation between the two groups (*χ* ^2 ^= 4.168, *P*=0.041).

### Elasticity evaluation of CBTs by SWE

Emax expressed in kPa and m/s was used to evaluate the intra-operator consistency of quantitative SWE. The correlation coefficient of Emax expressed in kPa and m/s was 0.940 (95% CI: 0.856-0.976) and 0.919 (95% CI: 0.804-0.966) for intra-operator consistency.

Emax distribution (kPa and m/s) of palpation hard CBTs was different from that of soft group (**Table 2**), Emax (kPa and m/s) were significantly higher in the palpation hard group than in the soft group (*Z*=2.572, *P*=0.009; *Z*=2.673, *P*=0.006). SDmax (kPa) were higher in the palpation hard group than in the soft group (*Z*=2.012, *P*=0.043); whereas SDmax distribution (m/s) had no significant difference between the two groups (*Z*=-0.396, *P*=0.722).

**Table 2:** Elasticity of CBTs on SWE with different manual palpation stiffnesses.

Manual palpation	N (%)	Emax (kPa)	Emax (m/s)	SDmax (kPa)	SDmax (m/s)
Soft	12(54.5)	42.5	3.2	22.4	1.1
Hard	10(45.5)	102.1	5.7	33.7	1.2
*Z*		2.572	2.673	2.012	-0.396
*P*		**0.009**	**0.006**	**0.043**	0.722

The difference is statistically significant in bold;

CBTs, carotid body tumors; Emax, the maximum elasticity; SDmax, standard deviation of the elasticity.

Emax distribution (kPa and m/s) and SDmax (kPa) were different in the three Shamblin types (χ^2 ^= 6.467, *P*=0.039; χ^2 ^= 6.110, *P*=0.047; χ^2 ^= 6.315, *P*=0.043), Emax (kPa and m/s) increased from shambling I to Shambling II and Shambling III; whereas the distribution of SDmax in m/s had no significant difference among the three Shamblin types (χ^2 ^= 1.663, *P*=0.435). (**Table 3**).

**Table 3:** Elasticity of CBTs on SWE with different Shamblin types.

Shamblin type	N (%)	Emax (kPa)	Emax (m/s)	SDmax (kPa)	SDmax (m/s)
Shamblin I	2(9.1)	14.4	1.9	9.1	0.8
Shamblin II	18(81.8)	75.6	4.8	27.5	1.2
Shamblin III	2(9.1)	137.0	7.2	43.4	1.4
χ^2^		6.467	6.110	6.315	1.663
*P*		**0.039**	**0.047**	**0.043**	0.435

The difference is statistically significant in bold;

CBTs, carotid body tumors; Emax, the maximum elasticity; SDmax, standard deviation of the maximum elasticity.

Emax distribution (kPa and m/s) of malignant CBTs were different from the benign ones, Emax (kPa and m/s) were significantly higher in the malignant CBTs than in the benign ones (*Z*=2.440; *P*=0.009; *Z*=2.442, *P*=0.009), whereas the distribution of SDmax (kPa and m/s) had no significant difference between the malignant and the benign groups (*Z*=1.867, *P*=0.069; *Z*=-1.792, *P*=0.085) (**Table 4**). Emax in kPa and m/s had the similar AUC value (AUC=0.947, 95%CI : 0.761-0.998, *P*=1.0000) with corresponding cut-off value of 124.9kPa and 5.9m/s in the prediction of malignant CBTs (**Table 5**). Emax in kPa with the cut-off value 124.9kPa showed a sensitivity of 100.0%, specificity of 94.7% and an accuracy of 95.5% (*Z*=8.500, *P*<0.0001); Emax in m/s with the cut-off value 5.9m/s showed a sensitivity of 100.0%, specificity of 89.5% and an accuracy of 90.9% in the prediction of malignant CBTs (*Z*=9.143, *P*<0.0001).

**Table 4:** Elasticity of malignant and benign CBTs on SWE.

Group	N (%)	Emax (kPa)	Emax (m/s)	SDmax (kPa)	SDmax (m/s)
Benign	19 (86.4)	58.9	3.9	25.4	1.1
Malignant	3 (13.6)	137.3	7.1	41.3	0.9
*Z*		2.440	2.442	1.867	-1.792
*P*		**0.009**	**0.009**	0.069	0.085

The difference is statistically significant in bold;

CBTs, carotid body tumors; Emax, the maximum elasticity; SDmax, standard deviation of the maximum elasticity.

**Table 5:** The performance of SWE in the prediction of malignant CBTs.

Elasticity	Cut-off	Sen (%)	Spe (%)	PPV (%)	NPV (%)	Acc (%)	AUC	*Z*	*P*
Emax(kPa)	124.9	100	94.7	75	100	95.5	0.947	8.500	<0.0001
Emax(m/s)	5.9	100	89.5	60	100	90.9	0.947	9.143	<0.0001

Sen, Sensitivity; Spe, Specificity; PPV, Positive predictive value; NPV, Negative predictive value; Acc, Accuracy; AUC, Area under the receiver operating characteristic curve.

## Discussion

CBTs are rare and usually benign PGL and the potential for malignant transformation. Chronic hypoxia is a risk factor for PGL including those who lived at high altitude, with increased risk particularly in women (17, 22). Mutations in succinate dehydrogenase subunit D (SDHD) are currently the leading cause of hereditary head and neck PGLs (>50%), followed by SDHB (20%-35%) (23). Higher altitudes may also facilitate the likelihood of early and multifocal tumor development in SDH mutation carrier (24). In the present study, two patients (12.5%) were brothers who came from plateau area with an altitude of 2261m, both with bilateral CBTs; the mean living altitude of the patients with bilateral CBTs (37.5%) was 1127.3m (range 52-2261.2m). 68% patients included in the study are male, this is in contrast to the literature (25, 26), it was probably due to the small sample size and select bias existed.

Like other characteristics of US examination, SWE results can vary according to the operators. This variability may be related to a measurement error produced by operator-applied transducer pressure, also termed preload (27). The present study showed that intra-operator consistency of quantitative SWE was highly reproducible with transducer gently placed on the neck during the inspection. With increasing types of elastography systems, there is not only operator-dependent, but also system-dependent variation. Although SWV and elastic modulus did not agree equally across different elastography systems, excellent intra- and inter-observer reproducibility without significant difference were reported and the accuracy was high and reliable with different elastography systems, yet absolute measurements from the different techniques should not be used interchangeably (28–30), because the cut-off value may varies due to the different equipment applied with various imaging parameter and the number of enrolled cases and proportion of malignant tumors.

Shamblin reported that a linear relationship between the tumor size and Shamblin group (19). Jasper also confirmed that maximum tumor dimension and tumor volumes correlated significantly with Shamblin classification (31). Although small tumors need not be Shamblin type I, large tumors are more likely to be Shamblin type II or III (32). Malignant CBTs was observed to be characterized by advanced Shamblin type and larger tumor size (33). In the present study, the size of CBTs was positively correlated with shambling type and Emax (kPa and m/s). Emax (kPa and m/s) increased from shambling I to Shambling II and Shambling III, and Emax (kPa and m/s) were significantly higher in the malignant CBTs than in the benign ones. Therefore, The CBTs with larger size and advanced Shamblin type were more likely to have higher elastic modulus and malignancy risk. 40% of the sclerosing variant of PGL had lymph node metastases indicating that sclerosing variant of PGL may have a more aggressive histological behavior (34), it is expected to identify such tumors by SWE so as to make the appropriate monitoring or treatment decision for patients. Emax (kPa and m/s) had the similar AUC value in the malignancy prediction in CBTs, this was reasonable because the elastic modulus was derived from the formula based on the SWV of the SWE. The pathological cause of the elasticity difference between benign and malignant CBTs was not explored due to the rarity of malignant CBTs, it may be related to the dense collagen fiber in the tumor.

Malignant tissue is more heterogeneous than benign one. SDmean shows the internal heterogeneity of the lesion and higher SDmean is linked to a higher risk of malignancy in breast tumors (35). In the present study, SDmax was measured and the results showed there was no significant difference in SDmax between malignant and benign CBTs, this may be probably because the ROIs were placed on the stiffest part of the CBTs, not on the entire lesions as in the literature (SDmean) (35). It has been confirmed that the most useful shear wave measure appears to be the stiffness within a ROI placed on the stiffest part of a saved image (36). Another study showed that evaluating Emax was simpler and took less time than assessing Emean, moreover, Emax was less affected by the depth or size of the lesion applying SWE system (10). Therefore, the stiffest area of the CBTs were selected as the ROI and Emax and SDmax were acquired in the present study. In future, selecting the entire lesion to measure the overall stiffness and comparing it with the maximum stiffness for the assessment of CBTs can be conducted.

This study has limitation. CBTs are relatively rare tumors. This was a single-center study, the numbers of CBTs included in the study were small, and only three malignant CBTs were included because of the rarity of malignant CBTs. Future studies of a larger series would be worthwhile to discriminate the malignancy from benign CBTs.

## Conclusions

In summary, quantitative analysis of SWE obtained the good performance in the elasticity assessment of CBTs. SWE could be a useful, quantitative and objective modality for the evaluation of the stiffness and is expected to be applied for the prediction of CBTs malignancy.

## Data availability statement

The raw data supporting the conclusions of this article will be made available by the authors, without undue reservation.

## Ethics statement

The studies involving human participants were reviewed and approved by the institutional review board of Peking Union Medical College Hospital. The patients/participants provided their written informed consent to participate in this study.

## Author contributions

Conceptualization and funding acquisition, JL; Conceptualization and manuscript review, BZ; Data curation, methodology, formal analysis and writing original draft, XZ; Resources, YZ. All authors contributed to the article and approved the submitted version.
